# Experimentally Verified Analytical Models of Piezoelectric Cantilevers in Different Design Configurations

**DOI:** 10.3390/s21206759

**Published:** 2021-10-12

**Authors:** Zdenek Machu, Ondrej Rubes, Oldrich Sevecek, Zdenek Hadas

**Affiliations:** 1 Laboratory of Energy Harvesting, Institute of Solid Mechanics, Mechatronics and Biomechanics, Faculty of Mechanical Engineering, Brno University of Technology, 616 69 Brno, Czech Republic; Ondrej.Rubes@vut.cz (O.R.); sevecek@fme.vutbr.cz (O.S.); hadas@fme.vutbr.cz (Z.H.); 2 Institute of Physics, Czech Academy of Sciences, 182 21 Prague, Czech Republic

**Keywords:** energy harvesting, vibrations, piezoelectric, analytical model, beam model, equivalent model, power prediction

## Abstract

This paper deals with analytical modelling of piezoelectric energy harvesting systems for generating useful electricity from ambient vibrations and comparing the usefulness of materials commonly used in designing such harvesters for energy harvesting applications. The kinetic energy harvesters have the potential to be used as an autonomous source of energy for wireless applications. Here in this paper, the considered energy harvesting device is designed as a piezoelectric cantilever beam with different piezoelectric materials in both bimorph and unimorph configurations. For both these configurations a single degree-of-freedom model of a kinematically excited cantilever with a full and partial electrode length respecting the dimensions of added tip mass is derived. The analytical model is based on Euler-Bernoulli beam theory and its output is successfully verified with available experimental results of piezoelectric energy harvesters in three different configurations. The electrical output of the derived model for the three different materials (PZT-5A, PZZN-PLZT and PVDF) and design configurations is in accordance with lab measurements which are presented in the paper. Therefore, this model can be used for predicting the amount of harvested power in a particular vibratory environment. Finally, the derived analytical model was used to compare the energy harvesting effectiveness of the three considered materials for both simple harmonic excitation and random vibrations of the corresponding harvesters. The comparison revealed that both PZT-5A and PZZN-PLZT are an excellent choice for energy harvesting purposes thanks to high electrical power output, whereas PVDF should be used only for sensing applications due to low harvested electrical power output.

## 1. Introduction

Energy harvesting is more than 20 years a hot topic in the field of wireless sensing [1] since it allows for converting various energy types from ambient sources into an electrical one. Although the amount of such harvested energy is usually small (tens of μW up to several mW), it can be used as a source of electrical power for modern, low power-consuming sensors that are typically used in wearable electronics and industrial applications [2] where powering using cables is not feasible (either due to a hazardous environment or complex setup). Piezoelectric kinetic energy harvesters in the form of a vibrating multilayer structure with piezoelectric layers [3] are commonly used in vibration energy harvesting applications, where the structure is excited by an ambient source of vibrations. The main task of kinetic energy harvesters is then to transform the mechanical energy of ambient vibrations, mainly those of machine frames or human body movement, into useful electrical energy by means of the direct piezoelectric phenomenon.

The main goal in the field of energy harvesting is to design a kinetic energy harvester which is capable to generate a sufficient amount of electrical energy in a particular vibratory environment [4] in order to power some other electronic equipment. However, each application has its requirements or limits for dimensions and weight of the harvester; the principle of energy harvesting can be used practically everywhere, for example in the field of medicine [5], wearables [6], portables [7], aircrafts [8], structural health monitoring of railways [9] or bridges [10].

It has been proved many times that for harvesting energy from ambient vibrations the kinematically excited cantilever beam is one of the most effective designs of a piezoelectric energy harvester. The fundamental and also the most important issue of this solution is the choice of a suitable piezoelectric material for effective electromechanical conversion. The review of commonly used piezoelectric materials and structures for energy harvesting purposes is summarized in publication [11], where it is shown that not only the material itself but also the intended operational mode significantly affects the amount of harvested power due to a great variation in piezoelectric coefficients. The highest piezoelectric coefficients (generally, the higher the coefficients, the higher the amount of harvested power) are provided by piezoceramic materials [12], especially those based on lead (PZT). As a non-toxic alternative, new lead-free piezoceramic materials have been developed which are based on multifunctional Perovskite [13] or structured layers made of Barium and Titanate [14]. Besides these piezoceramic materials which are inherently very brittle and stiff, there are also more flexible materials such as macro-fiber composites which are very promising in the area of strain energy harvesting [15] and piezopolymers which are summarized in review paper [16]. An example of a cantilever harvesting device based on a piezoelectric polymer (PVDF) is presented in paper [17] and the effectivity of PVDF in energy harvesting applications is nowadays widely discussed [6].

In conclusion, the two most important factors that determine the effectiveness of a vibrational energy harvesting device are the used piezoelectric material and the harvester’s geometry. Many recent works were concerned about the optimal harvester’s geometry for selected piezoelectric material, e.g., [18], but the effectivity of various piezoelectric materials has not been widely discussed yet. Both the selection of efficient piezoelectric material and suitable geometry of the harvester can be solved with an appropriate model of the piezoelectric resonator. For this reason, the presented paper is organized as follows. First, derivation of an analytical beam model of a kinematically excited piezoelectric cantilever in both bimorph/unimorph configurations which also respects the dimensions of used tip mass. This beam model is subsequently reduced to a single degree-of-freedom (DOF) system using the first mode shape function. Although, the derivation of a coupled electromechanical model was published several times, e.g., [19,20,21,22,23], here, we also show the effect of chosen mode shape function which is used in reducing the beam model into single DOF model. Then, the model is verified with 3 different experimental results. Finally, the main aim of this paper is to provide a methodology based on a verified model that can be used to compare the effectivity of materials commonly used in energy harvesting applications.

## 2. Model of Piezoelectric Vibration Energy Harvester

In order to harvest as much energy from vibrations as possible, it is paramount to properly design dimensions of the harvester and optimize its electrical impedance. This goal can easily be achieved with an analytical model which is able to predict electromechanical response of piezoelectric energy harvesters. Therefore, here in this paper a single DOF model of a kinematically excited cantilever in both bimorph/unimorph configurations is derived. This analytical model is based on Euler-Bernoulli (thin) beam theory and its output is compared with results from experiments conducted with three different piezoelectric harvesters described further in the following section. Then, the derived model is used in a comparative study to compare the piezoelectric materials used in the experiments in terms of energy harvesting efficiency.

### 2.1. Bimorph Cantilever Beam with Piezoelectric Layers in Series

**Figure 1:**
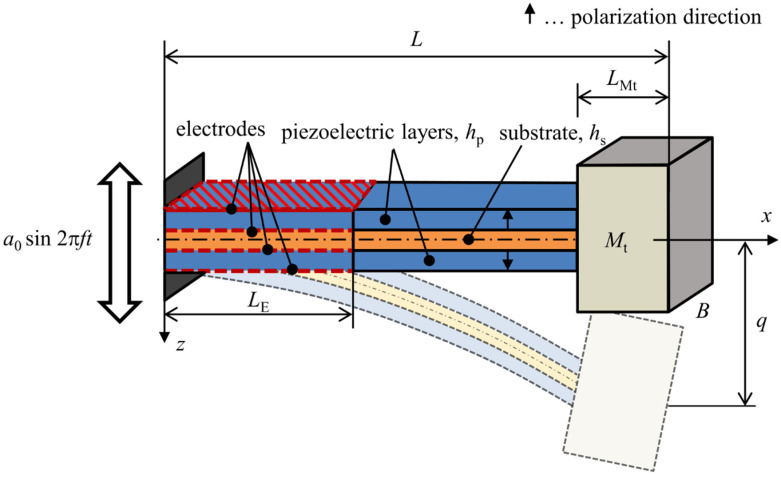
Geometric model of a piezoelectric bimorph in operational mode 31.

A piezoelectric cantilever beam harvester in a bimorph configuration is shown in Figure 1. This beam model with dimensions *L* × *B* × *H*, where *H* = 2 $h_{p}$ + $h_{s}$, is used for obtaining a single DOF analytical model. The clamped end is kinematically excited with a time-harmonic base acceleration *a*(*t*) from an external source of vibrations. Piezoelectric layers are in operational mode 31 (in-thickness polarization of piezoelectric layers and axial bending deformation of the harvester) whose polarization is denoted by arrow symbols in the figure. These layers have electrodes present over a region of dimensions $L_{E}$ × *B* which is mentioned further in the text as section *V*_E_; the remaining portion of piezoelectric layers is not polarized (mentioned as section *V*_R_), and thus is not affected by the piezoelectric effect. A tip mass $M_{t}$ of negligible rotary inertia is attached to the free end of the beam spanning over the length $L_{Mt}$—this section is denoted as *V*_R, Mt_. The bimorph model is reduced to a single DOF model which describes the movement of the bimorph’s free end *q* relative to the moving clamped end.

The assumptions of Euler-Bernoulli beam theory combined with those of the classical laminate theory concerning continuous strain throughout the layers of a multilayer structure imply that the strain $\varepsilon_{x}$ within bimorph’s layers can be expressed as

(1) $$\varepsilon_{x}=-\frac{\partial^{2}w}{\partial x^{2}}z,$$

where *w* is transverse displacement of the beam’s centerline relative to the movement of the excited clamped end. Since the beam is split into a section *V*_E_ which is affected by the piezoelectric effect, section *V*_R_ which is not affected by the piezoelectric effect and section *V*_R, Mt_ with distributed tip mass attached, the displacement needs to be a piecewise function defined as

(2) $$\;w\left(x,\;t\right)=\{\begin{array}{l} w_{1}\left(x,\;t\right)\;\mathrm{for}\;x\;\in \left[{0,\;L}_{E}\right]\\ w_{2}\left(x,\;t\right)\;\mathrm{for}\;x\;\in \left(L_{E}{,\;L\;-\;L}_{Mt}\right]\\ w_{3}\left(x,\;t\right)\;\mathrm{for}\;x\;\in \left({L\;-\;L}_{Mt},\;L\right] \end{array}.$$

For these functions it must hold that they are continuous and smooth at intersection points, i.e., they must satisfy following conditions:

(3) $$\begin{array}{c} w_{1}\left({x=L}_{E},\;t\right){=w}_{2}\left({x=L}_{E},\;t\right)\\ w_{2}\left({x=L\;-\;L}_{\mathrm{Mt}},\;t\right){=w}_{3}\left({x=L\;-\;L}_{\mathrm{Mt}},\;t\right)\\ \;\frac{\partial w_{1}}{\partial x}\left({x=L}_{E},\;t\right)=\frac{\partial w_{2}}{\partial x}\left({x=L}_{E},\;t\right)\\ \;\frac{\partial w_{2}}{\partial x}\left({x=L\;-\;L}_{\mathrm{Mt}},\;t\right)=\frac{\partial w_{3}}{\partial x}\left({x=L\;-\;L}_{\mathrm{Mt}},\;t\right) \end{array}.$$

The piezoelectric constitutive relations, which are required further in the derivation of the analytical model, take the following form for the uniaxial stress state [23]:

(4) $$\varepsilon_{x}{=s}_{11}^{E}\sigma_{x}{+d}_{31}E_{z},$$

(5) $$D_{z\;}{=d}_{31}\sigma_{x}+\epsilon_{33}^{T}E_{z},$$

where $\varepsilon_{x}$ represents normal strain, $s_{11}^{E}$ is mechanical compliance measured at constant electric field, $\sigma_{x}$ is normal stress, $d_{31}$ is the mode-31 component of the piezoelectric charge coefficient matrix, $E_{z}$ is the *z*-component of electric field intensity, $D_{z}$ is the *z*-component of electric flux density and $\epsilon_{33}^{T}$ is permittivity in direction of the polarization axis measured at constant mechanical stress. From Equation (4) the expression for the stress $\sigma_{x}$ is extracted as

(6) $$\sigma_{x}={\left(s_{11}^{E}\right)}^{-1}\varepsilon_{x}\;-\;{\left(s_{11}^{E}\right)}^{-1}d_{31}E_{z}\;.$$

Note that the reciprocal value of $s_{11}^{E}$ equals to the elastic modulus $Y_{p}$ of used piezoelectric material. Equation (6) can then be rewritten as

(7) $$\sigma_{x}{=Y}_{p}\varepsilon_{x}\;-\underset{e_{31}}{\underbrace{Y_{p}d_{31}}}E_{z}{=Y}_{p}\varepsilon_{x}{\;-\;e}_{31}E_{z}\;,$$

where $e_{31}$ is the piezoelectric modulus. Substituting (7) into (5) yields

(8) $$D_{z}=\underset{e_{31}}{\underbrace{d_{31}Y_{p}}}\varepsilon_{x}+\underset{\epsilon_{33}^{S}}{\underbrace{\left(\epsilon_{33}^{T}{\;-\;d}_{31}e_{31}\right)}}E_{z}{=e}_{31}\varepsilon_{x}+\epsilon_{33}^{S}E_{z},$$

where $\epsilon_{33}^{S}$ is the permittivity of used piezoelectric material measured at constant strain.

Piezoelectric materials are dielectrics and, as a consequence of the Gauss’ law, it holds that ∂$D_{z}$/∂*z* = 0 [24]. This implies that $D_{z}$ = const. Since the strain term in (8) changes linearly with the *z*-coordinate, we can use its mean value at the center of the *n*-th piezoelectric layer; the *z*-coordinate of the center of *n*-th layer is denoted as $z_{\mathrm{Tp}n}$. Next, the fundamentals of electricity, see e.g., [25], state that integration of $E_{z}$ over the thickness of a particular layer yields the voltage drop for the given layer. In order to make $D_{z}$ independent of the *z*-coordinate, $E_{z}$ must be a linear function of this coordinate with its mean value at the center of *n*-th layer given by

(9) $$E_{z}\left(z_{\mathrm{Tp}n}\right)=-\frac{U_{n}}{h_{p}}=-\frac{U}{{2h}_{p}}$$

where *U* is the magnitude of generated voltage drop and $h_{p}$ is the thickness of piezoelectric layers. By using Equations (1) and (9) in Equation (8) and by assuming that $D_{z}$ is a layer-wise function, one receives:

(10) $$D_{z}{=-e}_{31}z_{\mathrm{Tp}n}\frac{\partial^{2}w}{\partial x^{2}}\;-\;\frac{\epsilon_{33}^{S}}{{2h}_{p}}U.$$

By combining Equations (8) and (10), the following expression for $E_{z}$ is obtained:

(11) $$E_{z}=\frac{e_{31}}{\epsilon_{33}^{S}}\frac{\partial^{2}w}{\partial x^{2}}\left({z\;-\;z}_{\mathrm{Tp}n}\right)\;-\;\frac{U}{{2h}_{p}}.$$

According to [26], the electric current *I* generated by two in-series connected piezoelectric layers can be expressed as

(12) $$\;I=\frac{U}{R_{l}}=\frac{d}{dt}\left(\overset{}{\underset{A_{E}}{\iint }}D_{z}dxdy\right),$$

where the subscript $A_{E}$ denotes the area of electrodes and $R_{l}$ represents the connected resistance. Inserting (10) into Equation (12) yields

(13) $$\frac{U}{R_{l}}=\frac{d}{dt}\left[\overset{}{\underset{A_{E}}{\iint }}\left({-e}_{31}z_{\mathrm{Tp}n}\frac{\partial^{2}w}{\partial x^{2}}\;-\;\frac{\epsilon_{33}^{S}}{{2h}_{p}}U\right)dxdy\right].$$

Integrating terms in (13) leads to a following PDE which governs the electrical behavior of the considered bimorph

(14) $$C_{\mathrm{eq}}\frac{dU}{dt}+\frac{1}{R_{l}}U=\kappa {\frac{\partial^{2}w}{\partial x\partial t}|}_{{x=L}_{E}},$$

where $C_{\mathrm{eq}}$ denotes the bimorph’s equivalent capacitance defined as

(15) $$C_{\mathrm{eq}}=\epsilon_{33}^{S}\frac{{BL}_{E}}{{2h}_{p}},$$

and *κ* represents generic electromechanical coupling defined as

(16) $${\kappa =-Be}_{31}z_{Tp}.$$

As a next step, Equations (11) and (1) can be inserted into Equation (7) to obtain an expression for stress $\sigma_{x}$ within the polarized piezoelectric layers:

(17) $$\sigma_{x}={-Y}_{p}\frac{\partial^{2}w}{\partial x^{2}}z+\frac{e_{31}^{2}}{\epsilon_{33}^{S}}\frac{\partial^{2}w}{\partial x^{2}}\left(z_{\mathrm{Tp}n}\;-\;z\right)+\frac{e_{31}}{{2h}_{p}}U.\;$$

Within the other layers (non-polarized piezoelectric layers and the substrate) the stress obeys the Hooke’s law:

(18) $$\sigma_{x}{=-Y}_{n}\frac{\partial^{2}w}{\partial x^{2}}z\;,$$

where $Y_{n}$ denotes elastic modulus of the used piezoelectric material $Y_{p}$ or the substrate $Y_{s}$.

Total energy stored in the considered bimorph upon vibrations consists of kinetic energy $E_{k}$, strain energy $E_{p}$ and the work done by inertial forces due to kinematic excitation $W_{\mathrm{ext}}$. Kinetic energy of the considered beam can be written as

(19) $$\begin{array}{ll} E_{k} & =\frac{1}{2}\overset{}{\underset{V_{E}}{∭}}\left[\rho_{n}{\left(\frac{\partial w_{1}}{\partial t}\right)}^{2}\right]dV+\frac{1}{2}\overset{}{\underset{V_{R}}{∭}}\left[\rho_{n}{\left(\frac{\partial w_{2}}{\partial t}\right)}^{2}\right]dV+\frac{1}{2}\overset{}{\underset{V_{R,\;\mathrm{Mt}}}{∭}}\left[\left(\rho_{n}+\frac{M_{t}}{L_{\mathrm{Mt}}BH}\right){\left(\frac{\partial w_{3}}{\partial t}\right)}^{2}\right]dV\\ & =\frac{1}{2}\overset{L_{E}}{\underset{0}{\int }}\left[m^{*}{\left(\frac{\partial w_{1}}{\partial t}\right)}^{2}\right]dx+\frac{1}{2}\overset{{L\;-\;L}_{\mathrm{Mt}}}{\underset{L_{E}}{\int }}\left[m^{*}{\left(\frac{\partial w_{2}}{\partial t}\right)}^{2}\right]dx+\frac{1}{2}\overset{L}{\underset{{L\;-\;L}_{\mathrm{Mt}}}{\int }}\left[\left(m^{*}+\frac{M_{t}}{L_{\mathrm{Mt}}}\right){\left(\frac{\partial w_{3}}{\partial t}\right)}^{2}\right]dx, \end{array}$$

where $\rho_{n}$ is density of the *n*-th layer, $M_{t}$ is the attached tip mass and m^*^ is the bimorph’s mass per unit of its length defined as

(20) $$m^{*}=B\left(\rho_{s}h_{s}{+2\rho }_{p}h_{p}\right).$$

Strain energy stored in the bimorph can be expressed as

(21) $$E_{p}=\frac{1}{2}\overset{}{\underset{V_{E}}{∭}}\left[\varepsilon_{x}\sigma_{x}\right]dV+\frac{1}{2}\overset{}{\underset{V_{R}}{∭}}\left[Y_{n}\varepsilon_{x}^{2}\right]dV+\frac{1}{2}\overset{}{\underset{V_{R,\;\mathrm{Mt}}}{∭}}\left[Y_{n}\varepsilon_{x}^{2}\right]dV\;=\frac{1}{2}\overset{L_{E}}{\underset{0}{\int }}\left[J_{\mathrm{piezo}}^{*}{\left(\frac{\partial^{2}w_{1}}{\partial x^{2}}\right)}^{2}{\;-\;\kappa w}_{1}\frac{d\delta }{dx}\left({x\;-\;L}_{E}\right)U\right]dx+\frac{1}{2}\overset{{L\;-\;L}_{\mathrm{Mt}}}{\underset{L_{E}}{\int }}\left[J^{*}{\left(\frac{\partial^{2}w_{2}}{\partial x^{2}}\right)}^{2}\right]dx+\frac{1}{2}\overset{L}{\underset{{L\;-\;L}_{\mathrm{Mt}}}{\int }}\left[J^{*}{\left(\frac{\partial^{2}w_{3}}{\partial x^{2}}\right)}^{2}\right]dx\;,$$

where dδ/d*x* is the first derivative of Dirac’s delta function, $J_{\mathrm{piezo}}^{*}$ denotes bending stiffness of the beam section where the polarization of a piezoelectric material is considered (over the length $L_{E}$) defined as

(22) $$J_{\mathrm{piezo}}^{*}=\frac{1}{12}Y_{s}{Bh}_{s}^{3}{+2Y}_{p}\left[\frac{1}{12}{Bh}_{p}^{3}+{\left(\frac{h_{p}}{2}+\frac{h_{s}}{2}\right)}^{2}{Bh}_{p}\right]+\upsilon ,$$

where

(23) $$\upsilon =2\;\times \;\frac{1}{12}\frac{e_{31}^{2}}{\epsilon_{33}^{S}}{Bh}_{p}^{3}\;,$$

and J^*^ is bending stiffness of the non-polarized section of the beam (the rest of the beam outside the length $L_{E}$) defined as

(24) $$J^{*}=\frac{1}{12}Y_{s}{Bh}_{s}^{3}{+2Y}_{p}\left[\frac{1}{12}{Bh}_{p}^{3}+{\left(\frac{h_{p}}{2}+\frac{h_{s}}{2}\right)}^{2}{Bh}_{p}\right].$$

The work done by inertia forces due to kinematic excitation is defined as

(25) $$\begin{array}{ll} W_{\mathrm{ext}} & =-\overset{}{\underset{V_{E}}{∭}}\left[\rho_{n}a_{0}w_{1}\right]dV\;-\;\overset{}{\underset{V_{R}}{∭}}\left[\rho_{n}a_{0}w_{2}\right]dV-\overset{}{\underset{V_{R,\;\mathrm{Mt}}}{∭}}\left[\left(\rho_{n}+\frac{M_{t}}{L_{\mathrm{Mt}}BH}\right)a_{0}w_{3}\right]dV\\ & =-\overset{L_{E}}{\underset{0}{\int }}\left[m^{*}a_{0}w_{1}\right]dx\;-\;\overset{{L\;-\;L}_{\mathrm{Mt}}}{\underset{L_{E}}{\int }}\left[m^{*}a_{0}w_{2}\right]dx\;-\;\overset{L}{\underset{{L\;-\;L}_{\mathrm{Mt}}}{\int }}\left[\left(m^{*}+\frac{M_{t}}{L_{\mathrm{Mt}}}\right)a_{0}w_{3}\right]dx. \end{array}$$

Subsequently, Hamilton’s variational principle [27] is used to obtain equations of motion in the form of PDEs with a nonzero right-hand side. The equations of motion for the polarized portion of the beam and for the non-polarized portions of the beam take the following form:

(26) $$J_{\mathrm{piezo}}^{*}\frac{\partial^{4}w_{1}}{\partial x^{4}}{+m}^{*}\frac{\partial^{2}w_{1}}{\partial t^{2}}\;-\;\kappa \frac{d\delta }{dx}\left({x\;-\;L}_{E}\right){U=-m}^{*}a_{0},\;x\;\in \left[{0,\;L}_{E}\right]$$

(27) $$J^{*}\frac{\partial^{4}w_{2}}{\partial x^{4}}{+m}^{*}\frac{\partial^{2}w_{2}}{\partial t^{2}}{=-m}^{*}a_{0},\;x\;\in \left(L_{E}{,\;L\;-\;L}_{\mathrm{Mt}}\right]$$

(28) $$J^{*}\frac{\partial^{4}w_{3}}{\partial x^{4}}+\left(m^{*}+\frac{M_{t}}{L_{\mathrm{Mt}}}\right)\frac{\partial^{2}w_{3}}{\partial t^{2}}=-\left(m^{*}+\frac{M_{t}}{L_{\mathrm{Mt}}}\right)a_{0},\;x\;\in \left({L\;-\;L}_{\mathrm{Mt}},\;L\right]$$

Equations above, however, do not account for damping; therefore, they have to be extended with a damping term. Here, we shall consider the stiffness damping term from Rayleigh’s Damping theorem:

(29) $$J_{\mathrm{piezo}}^{*}\frac{\partial^{4}w_{1}}{\partial x^{4}}+\frac{{2b}_{r}}{\Omega_{1}}J_{\mathrm{piezo}}^{*}\frac{\partial^{5}w_{1}}{\partial x^{4}\partial t}{+m}^{*}\frac{\partial^{2}w_{1}}{\partial t^{2}}\;-\;\kappa \frac{d\delta }{dx}\left({x\;-\;L}_{E}\right){U=-m}^{*}a_{0},\;x\;\in \left[{0,\;L}_{E}\right]$$

(30) $$J^{*}\frac{\partial^{4}w_{2}}{\partial x^{4}}+\frac{{2b}_{r}}{\Omega_{1}}J^{*}\frac{\partial^{5}w_{2}}{\partial x^{4}\partial t}{+m}^{*}\frac{\partial^{2}w_{2}}{\partial t^{2}}{=-m}^{*}a_{0},\;x\;\in \left(L_{E}{,\;L\;-\;L}_{\mathrm{Mt}}\right]$$

(31) $$J^{*}\frac{\partial^{4}w_{3}}{\partial x^{4}}+\frac{{2b}_{r}}{\Omega_{1}}J^{*}\frac{\partial^{5}w_{3}}{\partial x^{4}\partial t}+\left(m^{*}+\frac{M_{t}}{L_{\mathrm{Mt}}}\right)\frac{\partial^{2}w_{3}}{\partial t^{2}}=-\left(m^{*}+\frac{M_{t}}{L_{\mathrm{Mt}}}\right)a_{0},\;x\;\in \left({L\;-\;L}_{\mathrm{Mt}},\;L\right]$$

where $b_{r}$ is the considered damping ratio and $\Omega_{1}$ is the value of the beam’s first eigenfrequency. Equations (29)–(31) together with (14) form a complete equation system which describes the electromechanical response of the considered bimorph.

However, this system of PDEs is actually not very effective to be used in modelling of energy harvesting devices because of its complexity, thus its transformation into a much simpler single DOF model is necessary. In the scope of vibrational energy harvesting applications, the beam is kinematically excited with frequencies very close or equal to the harvester’s first resonant frequency *f*_1,r_. This fact means that beam vibrations are composed mostly of the first vibrational mode and, as a consequence, the beam’s displacement relative to the base movement in all sections (*V*_E_, *V*_R_ and *V*_R, Mt_) can be written as

(32) $$\;w\left(x,\;t\right)\;\approx \;\phi_{1}\left(x\right)\eta_{1}\left(t\right),$$

where $\phi_{1}$ is the mode shape function of the first mode and $\eta_{1}$ is its modal coordinate. The shape function $\phi_{1}$ can be approximated with an arbitrary function that resembles the shape of the first bending mode. Although Erturk in [26] recommends using an approximative function which accounts for a tip mass at the beam’s free end, such a function is not appropriate for tip masses spanning over a finite length of the beam. Therefore, the following expression was chosen to simplify his approximative function into:

(33) $$\phi_{1}\left(x\right){=C}_{1}\cdot \left[\cos\frac{\lambda_{1}}{L}x\;-\;\cos h\frac{\lambda_{1}}{L}{x+\varsigma }_{1}\left(\sin\frac{\lambda_{1}}{L}x\;-\;\sin h\frac{\lambda_{1}}{L}x\right)\right],$$

where

(34) $$\varsigma_{1}=\frac{{\sin\lambda }_{1}{\;-\;\sin h\lambda }_{1}}{{\cos\lambda }_{1}{+\cos h\lambda }_{1}}.$$

The eigenvalue $\lambda_{1}$ is obtained as the first positive root of the following transcendental equation [26]

(35) $${1+\cos\lambda }_{1}{\cos h\lambda }_{1}=0.$$

Equation (33) accurately describes the first mode shape of a beam without a tip mass. Further in the paper it will be shown that simpler functions which deviate from the actual shape overestimate the beam’s stiffness and influence the calculated results.

The constant $C_{1}$ in (33) should be evaluated so that $\phi_{1}$ is mass-normalized to prevent numerical errors in further calculations of the model’s parameters, i.e., $\phi_{1}$ satisfies the following condition

(36) $$\overset{{L\;-\;L}_{\mathrm{Mt}}}{\underset{0}{\int }}\phi_{1}\left(x\right)m^{*}\phi_{1}\left(x\right)dx+\overset{L}{\underset{{L\;-\;L}_{\mathrm{Mt}}}{\int }}\phi_{1}\left(x\right)\left(m^{*}+\frac{M_{t}}{L_{\mathrm{Mt}}}\right)\phi_{1}\left(x\right)dx=1$$

Then, approximation (32) can be inserted into the equation system (14), (29)–(31) which can now be solved effectively using the Galerkin method [27], resulting into a much simpler equation system:

(37) $$M\frac{d^{2}\eta }{{dt}^{2}}+B\frac{d\eta }{dt}+K\eta +\theta U=F,$$

(38) $$C_{\mathrm{eq}}\frac{dU}{dt}+\frac{1}{R_{l}}U=\theta \frac{d\eta }{dt},$$

where

(39) $$\begin{array}{l} M=\overset{L-L_{\mathrm{Mt}}}{\underset{0}{\int }}\phi_{1}(x)m^{*}\phi_{1}(x)dx+\overset{L}{\underset{L-L_{\mathrm{Mt}}}{\int }}\phi_{1}(x)\left(m^{*}+\frac{M_{t}}{L_{\mathrm{Mt}}}\right)\phi_{1}(x)dx=1\\ K=J_{\mathrm{piezo}}^{*}\overset{L_{E}}{\underset{0}{\int }}{\left[\frac{d^{2}\phi_{1}(x)}{dx^{2}}\right]}^{2}dx+J^{*}\overset{L}{\underset{L_{E}}{\int }}{\left[\frac{d^{2}\phi_{1}(x)}{dx^{2}}\right]}^{2}dx=\Omega_{1}^{2}\\ B=2b_{r}\Omega_{1}\\ \theta ={\kappa \frac{d\phi_{1}}{dx}|}_{x=L_{E}}\\ F=-\left[m^{*}a_{0}\overset{L-L_{\mathrm{Mt}}}{\underset{0}{\int }}\phi_{1}(x)dx+\left(m^{*}+\frac{M_{t}}{L_{\mathrm{Mt}}}\right)a_{0}\overset{L}{\underset{L-L_{Mt}}{\int }}\phi_{1}(x)dx\right] \end{array}$$

#### 2.1.1. Effect of Chosen Mode Shape Function on Model Output

This section addresses the effect of the chosen approximative mode shape function $\phi_{1}$ on the model’s behavior. To this purpose, a reference configuration of a piezoelectric harvester with a significant tip mass is needed. This requirement is satisfied by a PZT-5A bimorph from a well-known work of Erturk and Inman [26].

**Figure 2:**
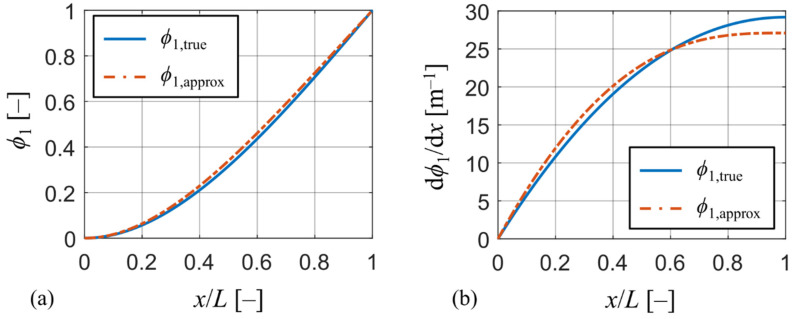
(**a**) Comparison between approximation and true mode shape; (**b**) comparison of slopes between approximation and true mode shape.

To analyze the influence of the chosen approximative function $\phi_{1}$ on the model’s output, the actual mode shape of the reference harvester is needed. To obtain the actual mode shape, a 3D numerical model of the reference harvester was created in commercial FE software ANSYS APDL made of approx. 1,000 SOLID186 higher-order elements. The actual mode shape denoted as $\phi_{1,\mathrm{true}}$ was obtained from a modal analysis of the model using the path post-processing tool. Both the actual mode shape $\phi_{1,\mathrm{true}}$ and the approximation $\phi_{1,\mathrm{approx}}$ defined by (33) normed to unity are plotted in Figure 2a. While mode shapes in the graph look almost identical, a much clearer distinction can be seen in Figure 2b by plotting their first derivatives with respect to *x* (the slope of the mode shape). Here, the actual mode shape $\phi_{1,\mathrm{true}}$ shows a much higher degree of compliance (higher value of d$\phi_{1}$/d*x*) at the beam’s free end, which is crucial for high power output. This increase in compliance at the beam’s free end is caused by the presence of the tip mass. Therefore, the presence of heavy tip masses at the beam’s free end causes an increase in beam’s compliance near its free end which cannot be accounted for using simpler approximative functions, such as polynomials. Using simpler approximative functions will lead to stiffer behavior of the beam model and result in higher resonant frequencies and underestimation of generated electrical power. Nevertheless, the errors in the model’s output by using (33) are not significant as demonstrated further in the paper.

#### 2.1.2. Single DOF Model of Bimorph Configuration

The system of Equations (37) and (38) is still not suitable for prediction of harvested power due to using the modal coordinate. For this reason, these equations are transformed from the modal coordinate into direct calculation of the relative movement of the bimorph’s free end *q* defined by (32) as

(40) $$\;q\left(t\right)=\phi_{1}\left(x=L\right)\eta_{1}\left(t\right),$$

Inserting (40) into (37) and (38) transforms the equation system into the sought single DOF model:

(41) $$M_{\mathrm{eff}}\frac{d^{2}q}{{dt}^{2}}{\;+\;B}_{\mathrm{eff}}\frac{dq}{dt}{\;+\;K}_{\mathrm{eff}}{q\;+\;\theta }_{\mathrm{eff}}{U\;=\;F}_{\mathrm{eff}}$$

(42) $$C_{\mathrm{eq}}\frac{dU}{dt}\;+\;\frac{1}{R_{l}}{U\;=\;\theta }_{\mathrm{eff}}\frac{dq}{dt}$$

where

(43) $$\begin{array}{l} M_{\mathrm{eff}}=\frac{M}{\phi_{1}^{2}\left(x=L\right)}=\frac{1}{\phi_{1}^{2}\left(x=L\right)}\\ K_{\mathrm{eff}}=\frac{K}{\phi_{1}^{2}\left(x=L\right)}=\frac{\Omega_{1}^{2}}{\phi_{1}^{2}\left(x=L\right)}\\ B_{\mathrm{eff}}=\frac{B}{\phi_{1}^{2}\left(x=L\right)}=\frac{{2b}_{r}\Omega_{1}}{\phi_{1}^{2}\left(x=L\right)}\\ \theta_{\mathrm{eff}}=\frac{\theta }{\phi_{1}\left(x=L\right)}=\frac{\kappa }{\phi_{1}\left(x=L\right)}{\frac{d\phi_{1}}{dx}|}_{{x=L}_{E}}\\ F_{\mathrm{eff}}=\frac{F}{\phi_{1}\left(x=L\right)} \end{array},$$

where capacitance $C_{\mathrm{eq}}$ is defined through Equation (15) and the connected resistive load $R_{l}$ represents the useful electrical load.

### 2.2. Modification of Single DOF Model for Unimorph Configuration

The model of a unimorph configuration of a piezoelectric harvester which considers only one piezoelectric layer is commonly used with piezoelectric polymers. The unimorph geometric model shares the same parameters to that of a bimorph shown in Figure 1. The single DOF model for the unimorph uses exactly the same equations that were derived for the bimorph, i.e., (41) and (42). Contrary to the bimorph, however, the major difference lies in fact that the bimorph’s neutral axis is coincident with its geometrical midplane, whereas this is not true in case of a unimorph. Therefore, the coefficients in (41) and (42) have to be re-defined to respect this fact.

First, the neutral axis of the unimorph $z_{N}$ is calculated as [28]

(44) $$z_{N}=\frac{Y_{s}z_{\mathrm{Ts}}h_{s}{\;+\;Y}_{p}z_{\mathrm{Tp}}h_{p}}{Y_{s}h_{s}{\;+\;Y}_{p}h_{p}}=\frac{1}{2}h_{s}h_{p}\frac{Y_{p}{\;-\;Y}_{s}}{Y_{s}h_{s}{\;+\;Y}_{p}h_{p}}.$$

Then, the coefficients in (41) and (42) are re-calculated with respect to the unimorph’s neutral axis $z_{N}$ using the same shape function $\phi_{1}$ as in (33). First, the mass coefficient $M_{\mathrm{eff}}$ is defined as

(45) $$M_{\mathrm{eff}}=\frac{\int_{0}^{{L\;-\;L}_{\mathrm{Mt}}}\phi_{1}\left(x\right)m^{*}\phi_{1}\left(x\right)dx\;+\;\int_{{L\;-\;L}_{\mathrm{Mt}}}^{L}\phi_{1}\left(x\right)\left(m^{*}\;+\;\frac{M_{t}}{L_{\mathrm{Mt}}}\right)\phi_{1}\left(x\right)dx}{\phi_{1}^{2}\left(x=L\right)},$$

where m^*^ changes to

(46) $$m^{*}=B\left(\rho_{s}h_{s}{\;+\;\rho }_{p}h_{p}\right).$$

Then, the stiffness coefficient $K_{\mathrm{eff}}$ changes to

(47) $$K_{\mathrm{eff}}=\frac{J_{\mathrm{piezo}}^{*}\int_{0}^{L_{E}}{\left(\frac{d^{2}\phi_{1}\left(x\right)}{{dx}^{2}}\right)}^{2}{dx\;+\;J}^{*}\int_{L_{E}}^{L}{\left(\frac{d^{2}\phi_{1}\left(x\right)}{{dx}^{2}}\right)}^{2}dx}{\phi_{1}^{2}\left(x=L\right)}\;,$$

for which the term $J_{\mathrm{piezo}}^{*}$ is defined as

(48) $$J_{\mathrm{piezo}}^{*}{=Y}_{s}B\left[\frac{1}{3}{\left(\frac{h_{s}{\;-\;h}_{p}}{2}\right)}^{3}+\frac{1}{3}{\left(\frac{h_{s}{\;+\;h}_{p}}{2}\right)}^{3}{\;-\;z}_{N}^{2}h_{s}\right]{\;+\;Y}_{p}B\left[\frac{1}{3}{\left(\frac{h_{s}{\;+\;h}_{p}}{2}\right)}^{3}\;-\;\frac{1}{3}{\left(\frac{h_{s}{\;-\;h}_{p}}{2}\right)}^{3}{\;-\;z}_{N}^{2}h_{p}\right]+\upsilon \;,$$

where

(49) $$\;\upsilon =\frac{e_{31}^{2}}{\epsilon_{33}^{S}}B\left[\frac{1}{3}{\left(\frac{h_{s}{\;+\;h}_{p}}{2}\right)}^{3}\;-\;\frac{1}{3}{\left(\frac{h_{s}{\;-\;h}_{p}}{2}\right)}^{3}{\;-\;z}_{\mathrm{Tp}}^{2}h_{p}\right]\;,$$

and J^*^ is defined as

(50) $${\;J}^{*}{=Y}_{s}B\left[\frac{1}{3}{\left(\frac{h_{s}{\;-\;h}_{p}}{2}\right)}^{3}+\;\frac{1}{3}{\left(\frac{h_{s}{\;+\;h}_{p}}{2}\right)}^{3}{\;-\;z}_{N}^{2}h_{s}\right]{\;+\;Y}_{p}B\left[\frac{1}{3}{\left(\frac{h_{s}{\;+\;h}_{p}}{2}\right)}^{3}\;-\;\frac{1}{3}{\left(\frac{h_{s}{\;-\;h}_{p}}{2}\right)}^{3}{\;-\;z}_{N}^{2}h_{p}\right]\;.$$

Next, the damping coefficient $B_{\mathrm{eff}}$ is defined as

(51) $$B_{\mathrm{eff}}=\frac{{2b}_{r}}{\Omega_{1}}K_{\mathrm{eff}}\;,$$

and the electromechanical coupling coefficient $\theta_{\mathrm{eff}}$ changes to

(52) $$\theta_{\mathrm{eff}}=\frac{\kappa }{\phi_{1}\left(x=L\right)}{\frac{d\phi_{1}}{dx}|}_{{x=L}_{E}}\;,$$

where *κ* is re-calculated with respect to the neutral axis $z_{N}$ as

(53) $$\;{\kappa =-e}_{31}\left(z_{\mathrm{Tp}}{\;-\;z}_{N}\right).$$

Then, the effective load $F_{\mathrm{eff}}$ is defined as

(54) $${\;F}_{\mathrm{eff}}=-\frac{m^{*}a_{0}\int_{0}^{{L\;-\;L}_{\mathrm{Mt}}}\phi_{1}\left(x\right)dx\;+\;\left(m^{*}\;+\;\frac{M_{t}}{L_{\mathrm{Mt}}}\right)a_{0}\int_{{L\;-\;L}_{\mathrm{Mt}}}^{L}\phi_{1}\left(x\right)dx}{\phi_{1}\left(x=L\right)}\;,$$

and the equivalent capacity $C_{\mathrm{eq}}$ is defined as

(55) $$C_{\mathrm{eq}}=\epsilon_{33}^{S}\frac{{BL}_{E}}{h_{p}}.$$

## 3. Verification of Analytical Model Based on Experimental Results

In this chapter, the derived single DOF model is verified for a time-harmonic kinematic excitation. Three different piezoelectric energy harvesters with known geometry and materials are analyzed and their measured responses are compared with the simulations of the derived single DOF model.

The first experiment is a well-known published work of Erturk and Inman [26] where the authors used a bimorph with PZT-5A piezoelectric material and electrodes spanning over the whole bimorph’s length, providing a linear dynamic response. The other two experiments included both bimorph and unimorph configurations of piezoelectric harvesters with a partial electrode length. Both these experiments were conducted in laboratories of Brno University of Technology. The first of these experiments used a bimorph made of PZZN-PLZT piezoceramic [6] which exerted a weak non-linear response in the frequency domain. The second experiment used a simple unimorph configuration for wearables with a thin PVDF layer. Geometrical parameters and material data of individual harvesters for both the piezoelectric layer and the substrate are summarized in Table 1 and Table 2, respectively. Data for the PZT-5A bimorph is extracted from [26], the PZZN-PLZT from [6] and the PVDF from [16].

### 3.1. PZT-5A Bimorph with a Full Electrode Length and a Linear Response

This experiment was described and published in detail in paper [26]. This experimental work has a very high impact and for this reason it was used in our analysis as an etalon for the other piezoelectric harvesters. The geometric model of this piezoelectric harvester is in accordance with the model in Figure 1. The bimorph’s piezoelectric layers were made of PZT-5A and the substrate was made of brass. It included electrodes covering the whole bimorph’s length for harvesting the generated charge. Geometric parameters of the bimorph and properties of used materials are summarized above in Table 1 and Table 2, respectively. This bimorph had an experimentally determined damping ratio $b_{r}$ = 0.027. Since the authors did not state a full description of the tip mass’ position and dimensions, it is assumed that the tip mass is located exactly at the bimorph’s free end with dimensions allowing for considering the tip mass as a point particle.

This harvester was subjected to a time-harmonic kinematic excitation with a varying forcing frequency *f*. The experiment mapped how amplitudes of generated electrical power and amplitudes of velocity of the bimorph’s free end change with a varying forcing frequency upon different values of connected resistive load. Furthermore, the experiment mapped how the peak values of generated electrical power vary with connected resistive load at a specific forcing frequency.

**Figure 3:**
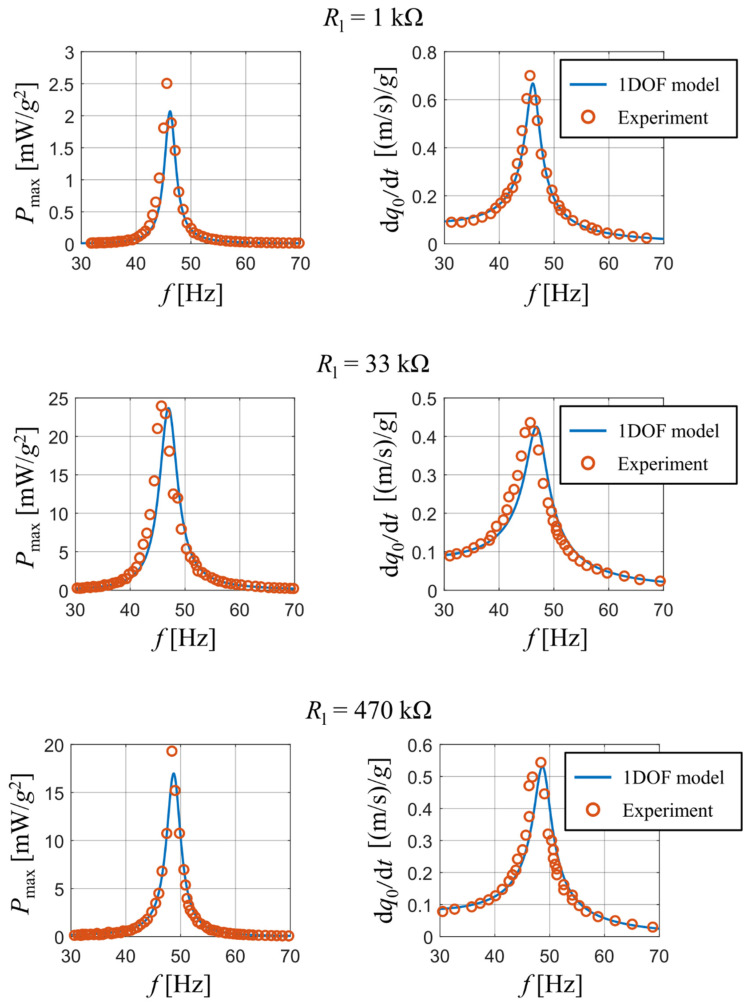
Comparison of electrical power and velocity of tip mass for both experimental results [26] and analytical model.

A comparison of published and measured results with the output of our analytical model is presented in Figure 3. The experiment tracked how the peak values of generated electrical power and the velocity amplitude at the beam’s free end d$q_{0}$/d*t* change with a varying forcing frequency. The results are displayed for three different values of used resistive load: 1 kΩ, 33 kΩ and 470 kΩ. The graphs show a good match between the output of the analytical model and the obtained experimental data for all three used resistive loads. Note that some discrepancies exist upon the first resonant frequency of the bimorph; this is mainly due to a steep gradient of calculated results near the first resonant frequency. The reader should also note that for resistive loads of 1 kΩ and 33 kΩ there is a slight difference in resonant frequencies between the real bimorph and the analytical single DOF model. This deviation is caused by the used approximative function $\phi_{1}$ which does not account for a concentrated tip mass at the beam’s free end. Therefore, as mentioned earlier, the used approximative function forces the beam to behave slightly stiffer and lowers the amount of generated electrical power.

**Figure 4:**
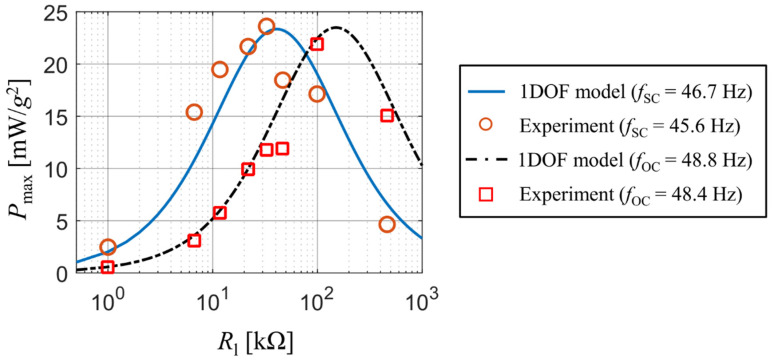
Peak power values as a function of resistive load upon excitation at short-circuit resonance frequency and the open-circuit resonance frequency.

The output of analytical model matches perfectly with the experimental results for both the short-circuit frequency *f*_SC_ and the open-circuit frequency *f*_OC_ of this coupled electromechanical system. The short-circuit and open-circuit frequency are the first resonant frequencies in case of $R_{l}$ = 0 and $R_{l}$ → ∞, respectively. The match of simulation results with the measured ones for various values of resistive load $R_{l}$ and kinematic excitation at both the short-circuit frequency *f*_SC_ and the open-circuit frequency *f*_OC_ is shown in Figure 4. Both states correspond with operations slightly below and above the resonance excitation for various values of resistive load, which determine the value of actual resonance frequency.

While in case of the open-circuit forcing frequency the results of the analytical single DOF model agree with the measured values, for the short-circuit case the calculated values from the single DOF model are slightly shifted towards higher values of resistive load. Also note that there are differences in both frequencies between the real bimorph and the single DOF model. While in case of the open-circuit frequency this difference is very small, for the short-circuit frequency this difference is notably larger and affects the value of optimal resistive load for which the generated electrical power reaches its peak value. This is caused by the used approximative function $\phi_{1}$ which causes the beam to behave stiffer. Nevertheless, this inaccuracy is negligible in terms of using analytical models for a rough prediction of the generated power when the system is excited by real vibrations.

### 3.2. PZNN-PLZT Bimorph with Partial Electrode Length and Weak Non-Linear Response

**Figure 5:**
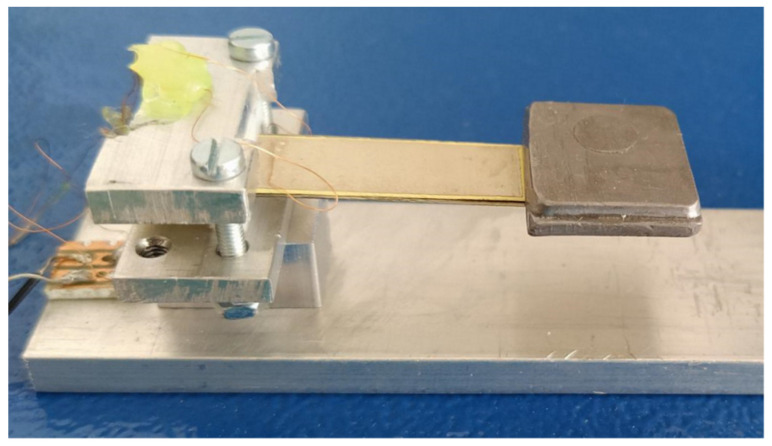
PZNN-PLZT bimorph used in experiment.

The experiment with PZNN-PLZT bimorph (see Figure 5) was conducted in a laboratory at Brno University of Technology with a partial electrode length (there are no electrodes under the tip mass). Geometrical parameters of this bimorph are summarized above in Table 1. The bimorph’s piezoelectric layers were made of PZNN-PLZT, which is in detail described in [6], and the substrate was made of a common steel shim. Properties of these materials are listed above in Table 2. Electrodes were made using a thin silver tape casting. Since the silver electrodes were substantially thinner than other layers, they were not accounted for in the calculation of single DOF model parameters due to their negligible effect on the net mass and the beam’s stiffness. The bimorph had an experimentally determined damping ratio $b_{r}$ = 0.025 via an impulse response in the short-circuit state.

The clamping of the used bimorph was kinematically excited at several forcing frequencies near the bimorph’s first resonant frequency with a constant acceleration amplitude $a_{0}$ = 0.1 g. The aim of this experiment was to track results, namely the RMS of generated voltage and RMS of velocity of the tip mass, at different excitation frequencies close to the bimorph’s first natural frequency. Also, the optimal resistive load was sought at which the bimorph generates maximal electrical power at its current first resonant frequency which slightly varies with changes in $R_{l}$.

**Figure 6:**
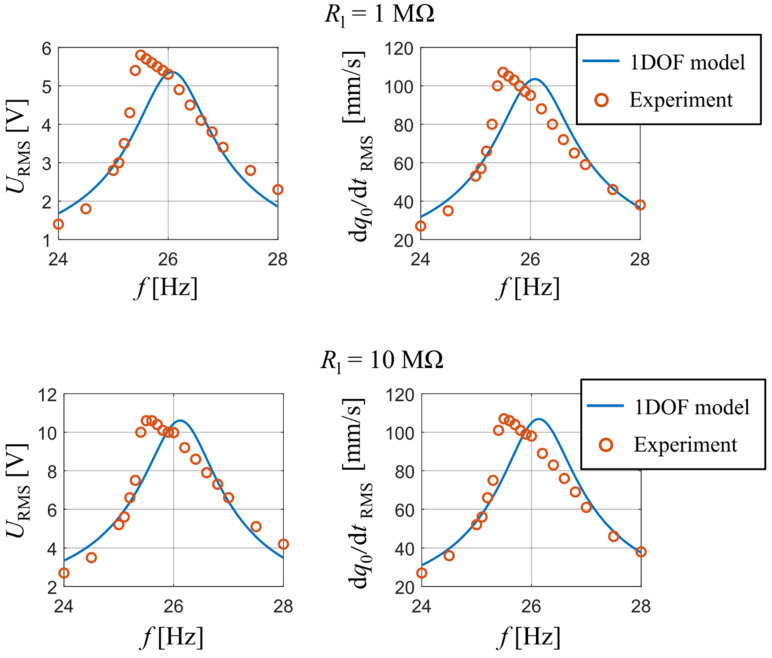
Comparison of generated voltage and velocity of harvester’s tip mass obtained from measurement and developed analytical model for *R*_l_ are 1 MΩ and 10 MΩ.

A comparison between the measured data and the calculated output of the analytical model is shown in Figure 6, namely the RMS values of output voltage and those of velocity of the bimorph’s free end as a function of forcing frequency. The results are displayed for two values of used resistive load: 1 MΩ and 10 MΩ. The measured data shows a weak non-linear softening dynamic behavior; however, the analytical single DOF model with linearized parameters still shows a very good degree of accuracy for both used resistive loads in terms of achieved amplitudes.

**Figure 7:**
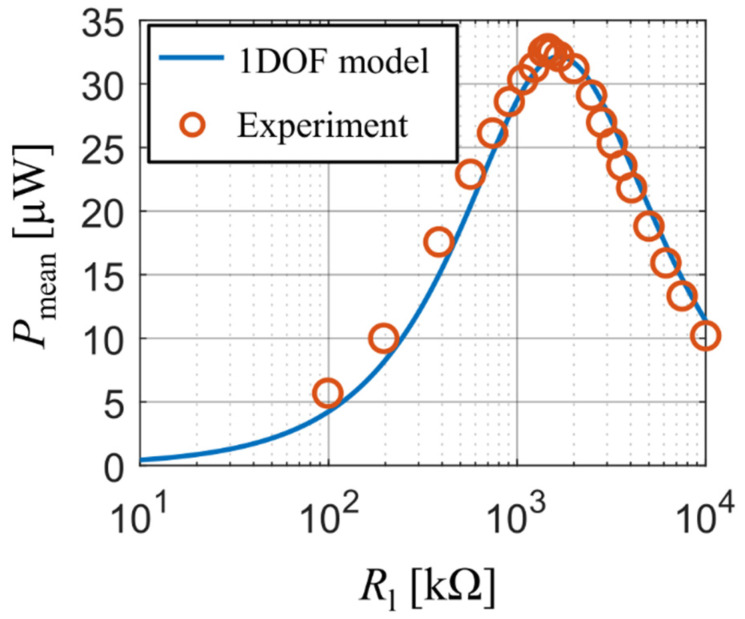
Power as function of resistive load upon excitation at actual resonant frequencies.

Our experiment also tracked the values of generated electrical power as a function of used resistive load. During the measurement, the forcing frequency was adjusted for each value of resistive load so that it matched the bimorph’s actual first resonant frequency. Both the analytical model and the experiment show (Figure 7) that the optimal resistive load is approx. 1.5 MΩ and, at the same time, also the maximal values of generated electrical power calculated with the single DOF model agree with experimental data at all used values of resistive load. The reader should note here that the curve from the analytical model is slightly shifted to higher values of $R_{l}$ which is, similarly as in the previous experiment, due to the used approximative function $\phi_{1}$, which makes the beam model behave slightly stiffer.

### 3.3. PVDF Unimorph with a Partial Electrode Length and a Linear Response

**Figure 8:**
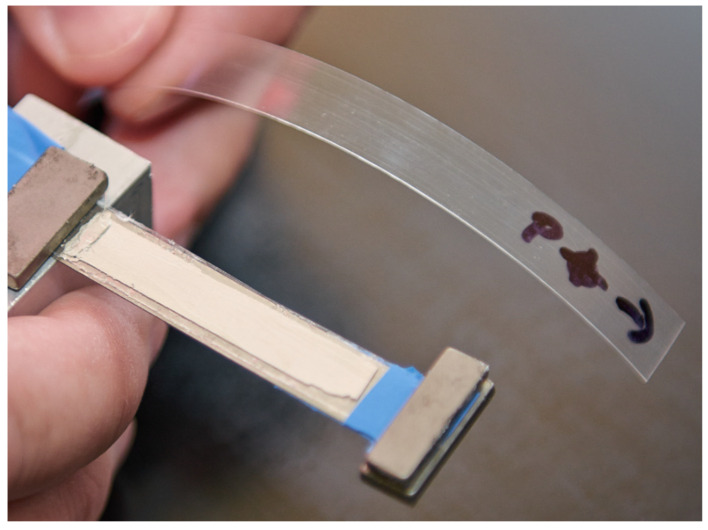
PVDF unimorph used in the experiment and a strip of PVDF foil used as the piezoelectric layer.

PVDF piezoelectric energy harvesters are very often presented as a suitable kinetic energy harvester [29] and for this reason the PVDF material was chosen for the last experiment, which was also conducted in a laboratory at Brno University of Technology. The PVDF foil is used in a unimorph configuration of a clamped cantilever with a partial electrode length shown in Figure 8. Parameters of this unimorph are listed above in Table 1. The unimorph’s piezoelectric layer is a PVDF foil and the substrate is a steel shim [30]. Properties of these materials are summarized above in Table 2. Electrodes were made using a thin silver tape casting. The silver electrodes were not accounted in the calculation of single DOF model parameters as in the previous model due to their negligible effect on the net mass and beam’s stiffness. The clamping of the used unimorph was kinematically excited at several forcing frequencies near the unimorph’s first natural frequency (*f*_1,r_ = 18.7 Hz) with a constant acceleration amplitude $a_{0}$ = 0.035 g. The unimorph had an experimentally determined damping ratio $b_{r}$ = 0.0065 via an analysis of impulse response in the short-circuit state.

**Figure 9:**
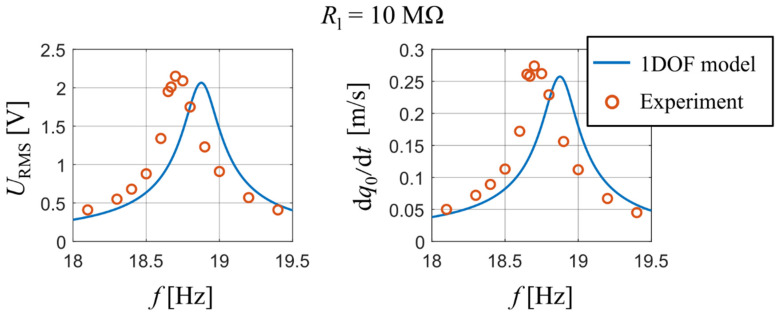
Comparison of output voltage and velocity of tip mass obtained from the measurement and by using the developed analytical model for *R*_l_ = 10 MΩ.

This experiment measured the RMS of output voltage and the amplitude of velocity of the tip mass at different forcing frequencies close to the unimorph’s first natural frequency. A comparison between the measured data and the calculated output of the analytical model is shown in Figure 9, namely the RMS values of output voltage *U* and amplitudes of velocity of the tip mass as a function of a forcing frequency for $R_{l}$ = 10 MΩ. One can see that the first resonant frequency of the analytical model is again slightly higher due to used approximative function $\phi_{1}$; nevertheless, the calculated values from the analytical model agree with the measured ones.

### 3.4. Single DOF Model Parameters of Considered Harvesters

The calculated parameters of individual harvesters which were used as input in analytical models are summarized in Table 3. The values of effective load $F_{\mathrm{eff}}$ were normalized with respect to 1 g of base acceleration.

The steady-state results calculated with the developed analytical single DOF model and parameters given in Table 3 showed an excellent agreement with all presented experiments. Combined with low usage of computer resources, the developed analytical model presents a simple and very effective tool for proper designing of piezoelectric harvesters. Moreover, the model can be used to simulate transient responses of the considered harvester (represented by its geometry and materials) to arbitrary time-dependent loads as demonstrated further in the text. For increased accuracy outside the vicinity of the harvester’s first resonant frequency, additional mode shapes ($\phi_{2}$, $\phi_{3}$, etc.) can be supplemented. Moreover, the single DOF model can easily be extended to support calculations of strain and stress levels within the beam’s layers to determine a maximal allowable load as shown in [31].

## 4. Comparison of Piezoelectric Materials for Kinetic Energy Harvesting Purposes

Since the analytical single DOF model of a piezoelectric harvester was successfully validated for both unimorph and bimorph configurations using the data from three different piezoelectric materials and experiments, the verified models of three harvesters will enable to determine the effectivity of used piezoelectric materials in different energy harvesting applications. Here, the three materials considered in the scope of this work (PZT-5A, PZZN-PLZT and PVDF) are compared in terms of harvested electrical power when subjected to harmonic vibrations (lab shaker) and in terms of harvested electrical energy when subjected to random vibrations (human body movement).

### 4.1. Harmonic Vibrations Case

To compare the output of harmonically excited piezoelectric harvesters made of different piezoelectric materials, their dynamic parameters must be similar, that is, their effective mass $M_{\mathrm{eff}}$ and eigenfrequency $f_{1}$. This can be done by changing dimensions of the considered harvesters; however, doing this will also lead to changes in the piezoelectric coupling coefficient and ultimately making the comparison invalid. To overcome this issue and maintain comparability, the volume of polarized piezoelectric materials was kept constant. The dimensions of polarized piezoelectric material in case of piezoceramic bimorphs were fixed at values $L_{E}$ × *B* × $h_{p}$ = 40 × 10 × 0.26 mm and in case of PVDF unimorph at $L_{E}$ × *B* × $h_{p}$ = 40 × 40 × 0.13 mm due to manufacturing limits of PVDF foils (these must be thin but can span over a large area). Then, to reduce the complexity of this optimizing task, the value of $L_{\mathrm{Mt}}$ was fixed at 5 mm and the thickness of the substrate $h_{s}$ was fixed at 0.15 mm (0.3 mm) in case of bimorphs (PVDF unimorph). Thus, the only parameters left for optimizing were the total length of the harvester *L* and and the tip mass $M_{t}$. These parameters were then tuned (see Table 4) to achieve values of $M_{\mathrm{eff}}$ and $f_{1}$ common to all three harvesters. Upon the study, the harvesters were forced with a time-harmonic base acceleration for various values of resistive load $R_{l}$ at their actual resonant frequencies.

**Figure 10:**
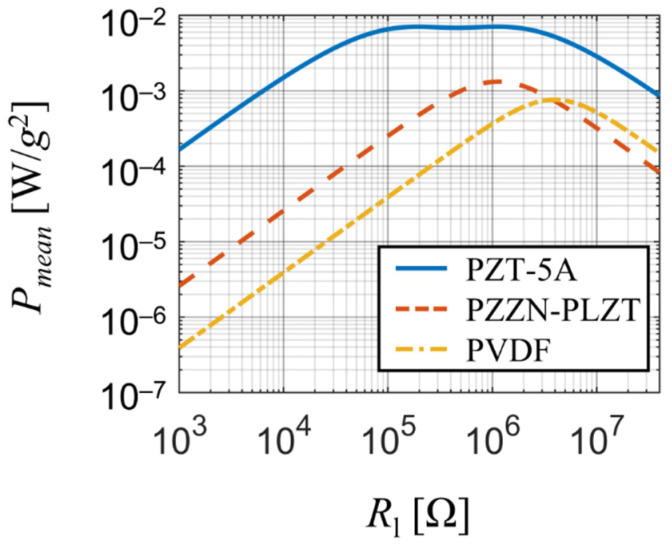
Comparison of harvested electrical power among the considered materials for various resistive loads upon simple harmonic forcing at the first resonant frequency.

The comparison (see Figure 10) revealed that PZT-5A is the best suitable material of the considered ones for energy harvesting purposes thanks to its high power output (several mW per 1 g of base acceleration) and a broad range of optimal resistive load (approx. 150 kΩ to 1.1 MΩ) due to its strong piezoelectric coupling. PZZN-PLZT is also suitable for energy harvesting applications since it offers high power output of about 1 mW per 1 g of base acceleration for resistive loads close to 1 MΩ. On the other hand, PVDF generates the least amount of power of the three materials and due to its very high optimal resistive load it is not sufficient for energy harvesting applications. Note that the strong piezoelectric coupling in case of PZT-5A harvester significantly damps the power output between the two optimal resistive loads which results in a local minimum surrounded by two local maxima.

### 4.2. Random Vibrations Case

**Figure 11:**
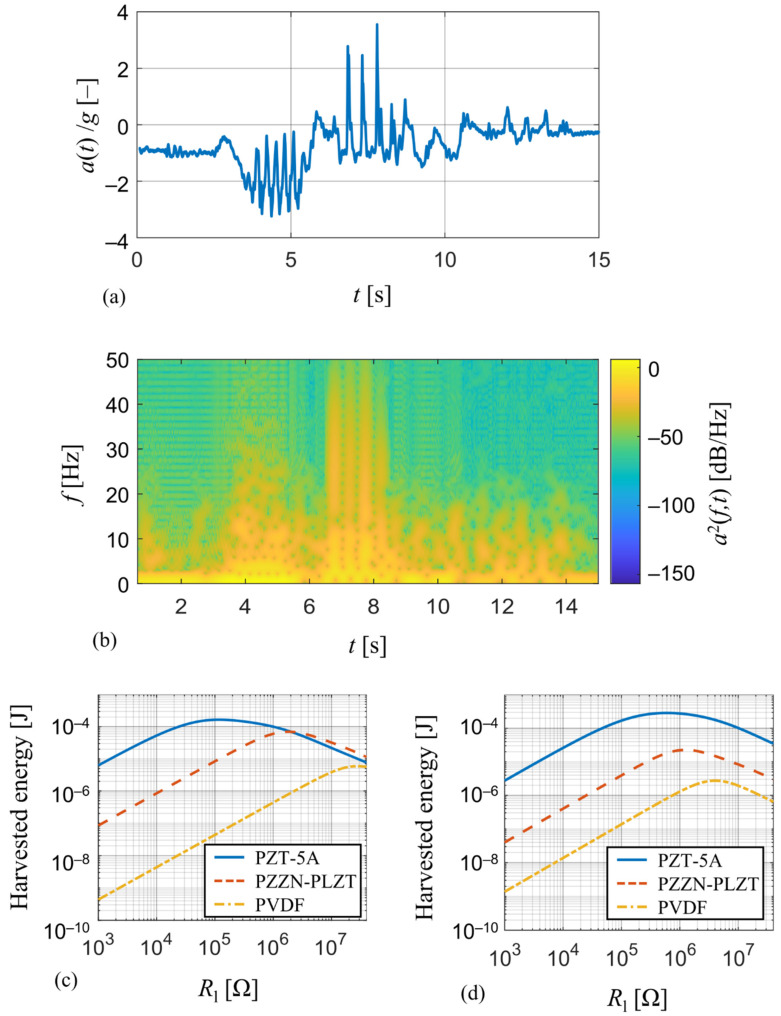
(**a**) Measured acceleration of a random movement of a human wearable and (**b**) its spectrogram; a comparison of harvested electrical energy from the human forearm movement among (**c**) the piezoelectric harvesters used in the experiments and (**d**) the tuned piezoelectric harvesters from Section 4.1 for different values of resistive load.

For this comparison, typical mechanical vibrations of a human forearm which are encountered in wearables applications were measured [32] and analyzed using the developed model. The measured time-course of acceleration *a*(*t*), see Figure 11a, is generated by a random movement of the forearm and can be thought of as a representative of random vibrations as can be seen from its spectrogram in Figure 11b. Therefore, it is perfect for the comparison of energy harvesting devices since a steady-state response, whose magnitude depends on how close the forcing frequency is to the harvester’s resonant frequency, will not occur.

The measured acceleration *a*(*t*) is used as input for a transient analysis of the derived single DOF analytical model, where the applied force is a function of acceleration data. This model of coupled electro-mechanical system is realized in Matlab Simulink simulation environment [33] and its aim is to track the amount of harvested electrical energy for various values of resistive load $R_{l}$. Simulation results of predicted harvested energy for this wearable operation for the harvesters used in the experiments and the tuned harvesters from Section 4.1 are shown in Figure 11c,d, respectively.

In case of unmodified harvesters used in the experiments (Figure 11c), the PZT-5A harvester is able to convert the most of mechanical energy (~0.17 mJ) among the three compared harvesters and has a low value of optimal resistive load (~118 kΩ). The energy output of PZZN-PLZT harvester (~0.07 mJ) is of the same order as the one of the PZT-5A harvester, however its much higher optimal resistive load (~1.82 MΩ) makes it less suitable for energy harvesting applications. On the contrary, the PVDF harvester is not suitable for energy harvesting applications at all due to very low energy output (~0.07 μJ), but it will find its use in sensing applications due to very high value of optimal resistive load (~28 MΩ). The same also applies to results in case of tuned harvesters (Figure 11d), where the tuned PZT-5A harvester once again shows that the energy harvesting properties of PZT-5A are far superior to those of PZZN-PLZT and PVDF. The increase in harvested electrical energy for the tuned PZT-5A harvester compared to the unmodified geometry is due to its lower resonant frequency which was reduced from 46.8 Hz to 31.1 Hz.

The results of both these comparisons showed that both PZT-5A and PZZN-PLZT piezoceramic harvesters are suitable for energy harvesting purposes, although operating at different values of resistive load, whereas the harvester based on piezoelectric polymer PVDF provides an insufficient energy harvesting system due to a very low amount of harvested energy. However this energy harvester should primarily be used in sensing applications [34].

## 5. Conclusions

The main aim of this paper was to compare the effectiveness of materials commonly used in energy harvesting operations using a single DOF model and at the same time analyze the effect of used mode shape function on simulation results. The single DOF model of a cantilever piezoelectric harvester in both bimorph and unimorph configurations was derived based on Euler-Bernoulli beam theory. Output of the model was confronted with available experimental data obtained from three different piezoelectric harvesters (PZT-5A bimorph, PZZN-PLZT bimorph and PVDF unimorph) and showed a good degree of accuracy. It is obvious that the presented model of an energy harvester can be used for various piezoelectric materials. Therefore, the developed single DOF analytical model represents a simple and very helpful tool for designing piezoceramic vibration energy harvesters. Moreover, it could easily be employed to check if a particular kinetic energy harvester provides sufficient output power for the intended application. Or inversely, the model could be used to design a piezoceramic harvester with optimized operational parameters and dimensions due to the model’s ability to predict the amount of harvested energy in particular operational conditions. Moreover, the developed model can easily be extended to support calculations of strain and stress levels within harvester’s layers for further assessments concerning strength and fatigue limits.

The model is primarily intended for operations of the harvester at frequencies where vibrations consist mostly of the first mode shape, since it offers the best operational conditions for energy harvesting (no strain nodes). If higher vibrational modes are of interest, the developed model can easily be extended by supplementing their respective shape functions and using the superposition principle. It was found that the quality of the used approximative function for the first mode shape affects the beam model’s stiffness in such a way so that simpler (less accurate) approximative functions force the beam model to behave stiffer, i.e., its resonant frequency being shifted to higher values. Also, the way how device layers and electrodes are assembled can also affect the stiffness of the system and could potentially result in a weak nonlinearity, which was observed in one of our experiments. Nevertheless, a typical assembly of layers and the approximative shape function used in this work (the shape of the beam’s first vibrational mode without a tip mass) still shows a good degree of accuracy.

The single DOF model itself poses as a very effective tool whose main advantages are low computer resources usage and the ability to calculate transient responses for arbitrary time-dependent loads. Both these features were employed in an energy harvesting effectivity comparison of the three materials used in the scope of this work. The materials comprised of PZT-5A, which is known nowadays to be one of the best materials for energy harvesting purposes, and PZZN-PLZT and PVDF which are used in our laboratory for designing vibration energy harvesting devices. The comparison was split into two parts with respect to forcing: case of simple harmonic vibrations and case of random vibrations. The case of simple harmonic vibrations was carried out so that the harvesters’ dimensions were tuned in order to achieve common value of seismic mass and resonant frequency for all three harvesters and at the same time the harvesters had same volume of polarized piezoelectric material. In case of random vibrations the harvesters were subjected to a non-harmonic and non-periodic vibrations typical for wearables applications. Results from both comparison cases showed that the piezoceramic harvesters (PZT-5A and PZZN-PLZT) are a perfect choice for energy harvesting applications, though geometry and electrical load must be optimized. On the contrary, the PVDF harvester is not suitable for energy harvesting purposes due to very low values of harvested energy despite many recent papers reporting the otherwise, and its potential lies in sensing applications.

## Figures and Tables

**Table 1:** Parameters of individual harvesters used in experiments.

Harvester Type (Configuration)	*L* [mm]	$L_{E}$ [mm]	$L_{\mathrm{Mt}}\;\;[\mathrm{mm}]$	*B* [mm]	$h_{s}\;\;[\mathrm{mm}]$	$h_{p}$ [mm]	$M_{t}\;\;[g]$
PZT-5A (bimorph)	50.8	50.8	–	31.8	0.14	0.26	12
PZZN-PLZT (bimorph)	40	25	15	10	0.1	0.2	10
PVDF (unimorph)	71.9	49.2	4	10	0.3	0.13	2.6

**Table 2:** Material properties of piezoelectric layers and substrates for each harvester used in experiments.

Harvester Type (Configuration)	Material	*Ρ* [kg/m^3^]	*Y* [GPa]	$d_{31}$ [C/N]	$\epsilon_{33}^{S}$ $/\epsilon_{0}\;\;[-]$
PZT-5A (bimorph)	PZT-5A	7800	66	–190 × 10^–12^	1500
	Brass shim	9000	105	–	–
PZZN-PLZT (bimorph)	PZNN-PLZT	7800	62.5	–195 × 10^–12^	1850
	Steel shim	7850	210	–	–
PVDF (unimorph)	PVDF	1760	2	–19 × 10^–12^	12
	Steel shim	7850	210	–	–

**Table 3:** Parameters of each piezoelectric harvester used in the analytical model.

Harvester Type	*M*_eff_ [g]	*B*_eff_ [Ns/m]	*K*_eff_ [N/m]	*F*_eff_ [N/*g*]	*θ*_eff_ [N/V]	*C*_eq_ [F]
PZT-5A	14.1	2.24 × 10^–1^	1218.10	1.51 × 10^–1^	2.20 × 10^–3^	4.12 × 10^–8^
PZZN-PLZT	6.10	5.13 × 10^–2^	164.56	7.90 × 10^–2^	6.03 × 10^–5^	3.65 × 10^–9^
PVDF	2.90	4.40 × 10^–3^	40.38	3.27 × 10^–2^	1.21 × 10^–6^	3.08 × 10^–10^

**Table 4:** Tuned dimensions of harvesters and their equivalent single DOF model parameters used in the comparison.

**Harvester Type** **(Configuration)**	***L*** **[mm]**	$L_{E}$ **[mm]**	$L_{\mathrm{Mt}}\;$ **[mm]**	***B*** **[mm]**	$h_{s}$ **[mm]**	$h_{p}$ **[mm]**	$M_{t}$ **[g]**
PZT-5A (bimorph)	68.8	40	5	10	0.15	0.26	3.67
PZZN-PLZT (bimorph)	54.3	40	5	10	0.15	0.26	3.99
PVDF (unimorph)	71.9	40	5	40	0.3	0.13	2.60
**Harvester Type** **(Configuration)**	$M_{\mathrm{eff}}\;$ **[g]**	$B_{\mathrm{eff}}$ **[Ns/m]**	$K_{\mathrm{eff}}$ **[N/m]**	$f_{1}\;$ **[Hz]**	$F_{\mathrm{eff}}/g\;$ **[N/1g]**	$\theta_{\mathrm{eff}}$ **[N/V]**	$C_{\mathrm{eq}}$ **[F]**
PZT-5A (bimorph)	4.21	4.45 × 10^–2^	161.25	31.13	5.02 × 10^–2^	5.14 × 10^–4^	1.02 × 10^–8^
PZZN-PLZT (bimorph)	4.21	4.20 × 10^–2^	161.05	31.13	4.75 × 10^–2^	6.36 × 10^–5^	4.50 × 10^–9^
PVDF (unimorph)	4.21	1.07 × 10^–2^	161.25	31.13	5.40 × 10^–2^	5.54 × 10^–6^	1.30 × 10^–9^

## References

[1] Roundy S., Wright P.K., Rabaey J. (2003). A study of low level vibrations as a power source for wireless sensor nodes. Comput. Commun..

[2] Mitcheson P.D., Yeatman E., Rao G.K., Holmes A.S., Green T. (2008). Energy Harvesting From Human and Machine Motion for Wireless Electronic Devices. Proc. IEEE.

[3] Roundy S., Wright P.K. (2004). A piezoelectric vibration based generator for wireless electronics. Smart Mater. Struct..

[4] Hadas Z., Smilek J., Rubes O., Fonseca L., Prunnila M., Peiner E. (2017). Analyses of electromagnetic and piezoelectric systems for efficient vibration energy harvesting. Smart Sensors, Actuators, and MEMS VIII.

[5] Gljušćić P., Zelenika S., Blažević D., Kamenar E. (2019). Kinetic energy harvesting for wearable medical sensors. Sensors.

[6] Bai Y., Tofel P., Hadas Z., Smilek J., Lošák P., Skarvada P., Macku R. (2018). Investigation of a cantilever structured piezoelectric energy harvester used for wearable devices with random vibration input. Mech. Syst. Signal Process..

[7] Paulo J., Gaspar P.D., Ao S.I., Gelman L., Hukins D. (2010). Review and future trend of energy harvesting methods for portable medical devices. WCE 2010—World Congress on Engineering 2010.

[8] Zelenika S., Hadas Z., Bader S., Becker T., Gljušćić P., Hlinka J., Janak L., Kamenar E., Ksica F., Kyratsi T. (2020). Energy Harvesting Technologies for Structural Health Monitoring of Airplane Components—A Review. Sensors.

[9] Duarte F., Ferreira A. (2017). Energy harvesting on railway tracks: State-of-the-art. Proc. Inst. Civ. Eng. Transp..

[10] Cahill P., Hanley C., Jaksic V., Mathewson A., Pakrashi V. Energy harvesting for monitoring bridges over their operational life. Proceedings of the 8th European Workshop on Structural Health Monitoring, EWSHM 2016.

[11] Bowen C.R., Kim H.A., Weaver P.M., Dunn S. (2014). Piezoelectric and ferroelectric materials and structures for energy harvesting applications. Energy Environ. Sci..

[12] Panda P.K., Sahoo B. (2015). PZT to lead free piezo ceramics: A review. Ferroelectrics.

[13] Bai Y., Tofel P., Palosaari J., Jantunen H., Juuti J. (2017). A Game Changer: A Multifunctional Perovskite Exhibiting Giant Ferroelectricity and Narrow Bandgap with Potential Application in a Truly Monolithic Multienergy Harvester or Sensor. Adv. Mater..

[14] Tofel P., Machu Z., Chlup Z., Hadraba H., Drdlik D., Sevecek O., Majer Z., Holcman V., Hadas Z. (2019). Novel layered architecture based on Al_2_O_3_/ZrO_2_/BaTiO_3_ for SMART piezoceramic electromechanical converters. Eur. Phys. J. Spec. Top..

[15] Pozzi M., Canziani A., Durazo-Cardenas I., Zhu M., Kundu T. (2012). Experimental characterisation of macro fibre composites and monolithic piezoelectric transducers for strain energy harvesting. Smart Structures (NDE).

[16] Sappati K.K., Bhadra S. (2018). Piezoelectric polymer and paper substrates: A review. Sensors.

[17] Song J., Zhao G., Li B., Wang J. (2017). Design optimization of PVDF-based piezoelectric energy harvesters. Heliyon.

[18] Kim M., Dugundji J., Wardle B.L. (2015). Efficiency of piezoelectric mechanical vibration energy harvesting. Smart Mater. Struct..

[19] Erturk A., Inman D.J. (2008). Issues in mathematical modeling of piezoelectric energy harvesters. Smart Mater. Struct. Smart Mater. Struct.

[20] Liao Y., Liang J. (2018). Maximum power, optimal load, and impedance analysis of piezoelectric vibration energy harvesters. Smart Mater. Struct..

[21] Li X., Upadrashta D., Yu K., Yang Y. (2018). Sandwich piezoelectric energy harvester: Analytical modeling and experimental validation. Energy Convers. Manag..

[22] Mendonca L.S., Martins L.T., Radecker M., Bisogno F., Killat D. (2019). Normalized Modeling of Piezoelectric Energy Harvester Based on Equivalence Transformation and Unit-Less Parameters. J. Microelectromech. Syst..

[23] Machů Z., Ševeček O., Hadas Z., Kotoul M. (2020). Modeling of electromechanical response and fracture resistance of multilayer piezoelectric energy harvester with residual stresses. J. Intell. Mater. Syst. Struct..

[24] Meitzler A. (1988). 176-1987 IEEE Standard on Piezoelectricity.

[25] Halliday D., Resnick R., Walker J. (2010). Fundamentals of Physics Extended.

[26] Erturk A., Inman D.J. (2009). An experimentally validated bimorph cantilever model for piezoelectric energy harvesting from base excitations. Smart Mater. Struct..

[27] Reddy J.N. (2017). Energy Principles and Variational Methods in Applied Mechanics.

[28] Flores-Domínguez M. (2014). Modeling of the Bending Stiffness of a Bimaterial Beam by the Approximation of One-Dimensional of Laminated Theory. J. Eng. Res. Appl..

[29] Zhao J., You Z. (2014). Models for 31-Mode PVDF Energy Harvester for Wearable Applications. Sci. World J..

[30] Hadas Z., Rubes O., Tofel P., Machu Z., Riha D., Sevecek O., Kastyl J., Sobola D., Castkova K. Piezoelectric PVDF Elements and Systems for Mechanical Engineering Applications. Proceedings of the 2020 19th International Conference on Mechatronics—Mechatronika (ME).

[31] Rubes O., Machu Z., Sevecek O., Hadas Z. (2020). Crack Protective Layered Architecture of Lead-Free Piezoelectric Energy Harvester in Bistable Configuration. Sensors.

[32] Rubes O., Hadas Z. Design and Simulation of Bistable Piezoceramic Cantilever for Energy Harvesting from Slow Swinging Movement. Proceedings of the 2018 IEEE 18th International Conference on Power Electronics and Motion Control, PEMC 2018.

[33] Rubes O., Brablc M., Hadas Z. (2019). Nonlinear vibration energy harvester: Design and oscillating stability analyses. Mech. Syst. Signal Process..

[34] Fitzgerald P.C., Malekjafarian A., Bhowmik B., Prendergast L.J., Cahill P., Kim C.-W., Hazra B., Pakrashi V., Obrien E.J. (2019). Scour Damage Detection and Structural Health Monitoring of a Laboratory-Scaled Bridge Using a Vibration Energy Harvesting Device. Sensors.

